# Philadelphia Horseshow Personals

**Published:** 1901-07

**Authors:** 


### PHILADELPHIA HORSESHOW PERSONALS.

Veterinary dentist Haley sighed many times, while watching the movements of the horses in the ring, that the owners, to his keen eye, had neglected having their teeth properly dressed.

Dr. A. F. Schrieber was noted amidst the parade of people around the tan bark.

Resident veterinarian Corman, of the University of Pennsylvania, did not find all the attractions in the ring ; there were others around the boardwalk.

Dr. Oscar Seeley, of Philadelphia, was a winner of a ribbon in the saddle class of St. Martin's-on-the-Green.

Dr. S. D. Larzelere, of Jenkintown, was one of the Decoration Day visitors and much interested in the heavy harness classes.

Dr. E. W. Powell, of the class of 1890, of the Veterinary Department of the University of Pennslyvania, spent a day at the show.

Dr. S. J. J. Harger, of Philadelphia, spent several days at the show, but found very few attractions outside the tan bark the first three days.

